# An NLP tool for data extraction from electronic health records: COVID-19 mortalities and comorbidities

**DOI:** 10.3389/fpubh.2022.1070870

**Published:** 2022-12-01

**Authors:** Sana S. BuHamra, Abdullah N. Almutairi, Abdullah K. Buhamrah, Sabah H. Almadani, Yusuf A. Alibrahim

**Affiliations:** ^1^Department of Information Science, Kuwait University, Kuwait City, Kuwait; ^2^Surgery Department, Al-Adan Hospital, Al Ahmadi, Kuwait; ^3^Department of Medical Imaging, Faculty of Medicine, University of Toronto, Toronto, ON, Canada

**Keywords:** natural language processing, text mining, information extraction, SARS-CoV-2, mortality, decision tree, prediction

## Abstract

**Background:**

The high infection rate, severe symptoms, and evolving aspects of the COVID-19 pandemic provide challenges for a variety of medical systems around the world. Automatic information retrieval from unstructured text is greatly aided by Natural Language Processing (NLP), the primary approach taken in this field. This study addresses COVID-19 mortality data from the intensive care unit (ICU) in Kuwait during the first 18 months of the pandemic. A key goal is to extract and classify the primary and intermediate causes of death from electronic health records (EHRs) in a timely way. In addition, comorbid conditions or concurrent diseases were retrieved and analyzed in relation to a variety of causes of mortality.

**Method:**

An NLP system using the Python programming language is constructed to automate the process of extracting primary and secondary causes of death, as well as comorbidities. The system is capable of handling inaccurate and messy data, this includes inadequate formats, spelling mistakes and mispositioned information. A machine learning decision trees method is used to classify the causes of death.

**Results:**

For 54.8% of the 1691 ICU patients we studied, septic shock or sepsis-related multiorgan failure was the leading cause of mortality. About three-quarters of patients die from acute respiratory distress syndrome (ARDS), a common intermediate cause of death. An arrhythmia (AF) disorder was determined to be the strongest predictor of intermediate cause of death, whether caused by ARDS or other causes.

**Conclusion:**

We created an NLP system to automate the extraction of causes of death and comorbidities from EHRs. Our method processes messy and erroneous data and classifies the primary and intermediate causes of death of COVID-19 patients. We advocate arranging the EHR with well-defined sections and menu-driven options to reduce incorrect forms.

## Introduction

The COVID-19 pandemic has had a significant impact on how and where healthcare is delivered effectively and efficiently. During the pandemic, the need for novel and current technologies arise to assist in predicting clinical outcomes in critical time with the high overflow of patients. Clinical (text) notes constitute a major source of medical data and are rarely used to their full capacity, even though they include a wealth of subjective information. Prior to electronic health records (EHRs), practitioners had to manually collect data from clinical notes, which was costly and difficult to scale up. Despite the expanding volumes of healthcare data, Kong (1) claims that over 80% of text, image, signal, and other medical data collections remain unstructured and unused. One main goal in medical research is to use EHRs to extract and analyze well-structured data. Many methods were devised and evaluated using EHRs for detecting patients with known risk factors for consequences such as stroke and significant bleeding (2), as well as investigating the difficulties of decoding and comprehending clinical narratives (3). Natural language processing (NLP) can expedite diagnosis and care to patients who are most vulnerable during pandemics by using textual data from medical records. According to Zhou et al. (4), only NLP can extract information about a patient's family history from free-text clinical papers. The researchers employed word embeddings and a Convolutional Neural Network (CNN) to recognize International Classification of Diseases (ICD-10) diagnostic codes in discharge notes and outperformed current methods with little data preparation (5).

Artificial Intelligence (AI) and Machine Learning (ML) technologies including NLP can be used to aid in the diagnosis and treatment of individuals suffering from acute and chronic diseases during the COVID-19 pandemic. DeCapprio et al. (6) used medical records that had already been made public as COVID-19 proxies (pneumonia, influenza, acute bronchitis, and upper respiratory illnesses). Zoabi et al. (7) came up with a machine learning decision tree model that predicts a positive COVID-19 infection in an RT-PCR test during the first month of the pandemic. Izquierdo et al. (8) used a mix of traditional epidemiological methods, NLP, and ML predictive modeling to find out what symptoms COVID-19 patients have that make them likely to be admitted to the ICU. Guan et al. (9) employed simple-tree XGBoost to identify high-risk COVID-19 cases and assessed how much faster causes of death may be identified using minimally preprocessed notes.

This study intends to construct an NLP system to automate the extraction of primary and secondary causes of death, as well as comorbidities, from the mortality EHRs of COVID-19 patients admitted to the ICU in Kuwait during the pandemic. Since many of the free-text notes were inadequately formatted, contained spelling mistakes and were placed in the wrong field, acquiring sufficient and reliable data was the largest hurdle. In fact, the causes of death in most records in our data were not expressed precisely nor was in the correct field although the EHRs file is mortality specific.

Other work in the literature used available clean EHRs for their analysis. However, EHRs may sometimes be inaccurate and noisy due to them being compiled under extreme pressures of time and manpower due to the large influx of patients with critical cases, such as the case during the pandemic. EHRs need to be first corrected and cleaned to be used for proper analysis or be used in medical systems such the Unified Medical Language System (UMLS) and SNOMED CT. Otherwise, a significant amount of information will be lost.

To correct the EHRs we used physicians as the domain knowledge experts to understand and extract the common mistakes in the EHRs that were done by their fellow physicians. Their knowledge and findings were converted to a Python language code to automate cleaning and fixing the data in the EHRs. Also, the Python code used the domain expert knowledge to distinguish between acute diseases and causes of death in some circumstances. In addition, the causes of death were classified to a direct cause or a related one. Comorbidities were used as an important factor in analyzing the cause of death. This will offer precise information on the casuality and spectrum of comorbidities in fatal instances, allowing for an accurate evaluation of COVID-19's hazardous nature. Finally, we have utilized a decision tree-based model to predict death due to ARDS or other complications. These findings can assist healthcare systems to plan for the spread of future pandemics and identify groups at risk.

## Methods

### The data

Data on COVID-19 mortalities were retrieved from Jaber Hospital's mortality Electronic Health Records (EHR) for all patients admitted to the ICU between March 7, 2020, and August 19, 2021, and death reported between March 7, 2020, and August 27, 2021. The data set contains 1691 cases after excluding 12 children (<17 years old) and 46 with no data entries. The monthly total death rate in Kuwait is depicted in Worldometer cite (10). On the final day of data collection for this study, the total number of COVID-19 deaths was reported to be 2415; thus, our sample size covers 70% (1691/2415) of the COVID-19 mortality population. We also covered all death peaks and pandemic main waves during this time.

Initially, the data was extracted as a pdf file and then converted to an Excel spreadsheet. Patients' demographics (age, gender, and residency), date of ICU admission, date of death, reasons for admission, admission diagnosis, final diagnosis, cause of death, brief history, brief summary, and contributing factors are all included in each record. To ensure confidentiality, all data was anonymized and all patient identifiers were removed. Additional data cleansing were also performed to ensure data accuracy.

### Creating corpus of terminologies

The data sheet obtained from EHR has mainly eleven columns: date of admission to ICU, date of death, age at death, admission diagnosis, reason for admission, final diagnosis, cause of death (COD), brief history, brief summary, and contributing factors. Except for the first three, all remaining columns are text features.

The underlying cause of death, such as “COVID-19” or “COVID 19 pneumonia,” was listed in the COD column in many records, whereas the primary/intermediate causes were found explicitly or indirectly in the brief summary or brief history columns. Furthermore, there were two major flaws in the free-text notes in the mortality EHR. The first issue is that many terminologies have misspellings or improper forms. For example, multiorgan failure is referred to as “Multi-Organs” or “Multiorgan Failure.” The second issue is inconsistency in the reporting of text notes. The causes of death are not always listed in the data columns that you would expect. Comorbidities, on the other hand, are not consistently included in the list of contributing factors. As a result, we are unable to use the existing NLP tools or Unified Medical Language System (UMLS). We had to develop our own system to extract concepts, knowledge, and relationships from the mortality EHR at hand.

#### CODs and comorbidity glossary tables

Our strategy is to extract the causes of death (COD) and comorbidities/diseases by using NLP techniques such as a bag-of-word (BoW) model. The BoW model will be applied on each column to extract all terms and phrases that represent the CODs and comorbidities for each patient. The model achieves this by tokenizing all text columns in the data sheet and creating a case/term occurrence matrix where each row represents a patient's case and each column represents each medical term relating to a cause of death or comorbidity, all other word tokens will be omitted. The cells of the matrix will contain a 0 or 1 representing the occurrence or absence of the term from the case. The terms related to cause of death will be categorized to three stages similar to the fashion of death certificates. These stages are the primary, intermediate and the underlying cause (which led to the intermediate).

The list of primary causes of death, according to WHO guidelines, denotes the condition (injury, complication, or disease) that directly preceded death. WHO issued an updated International Classification of Diseases (ICD) and health-related problems to accommodate COVID-19-related death complications (11). The condition(s) that led to the primary COD are reflected in the intermediate COD. Multiple complications contributing to the intermediate COD were identified in the majority of COVID-19 decedents in the ICU in this study. Additionally, COVID-19 pneumonia was the most frequently encountered underlying cause in those ICU cases, resulting in an intermediate stage of complication.

**Figure 1:**
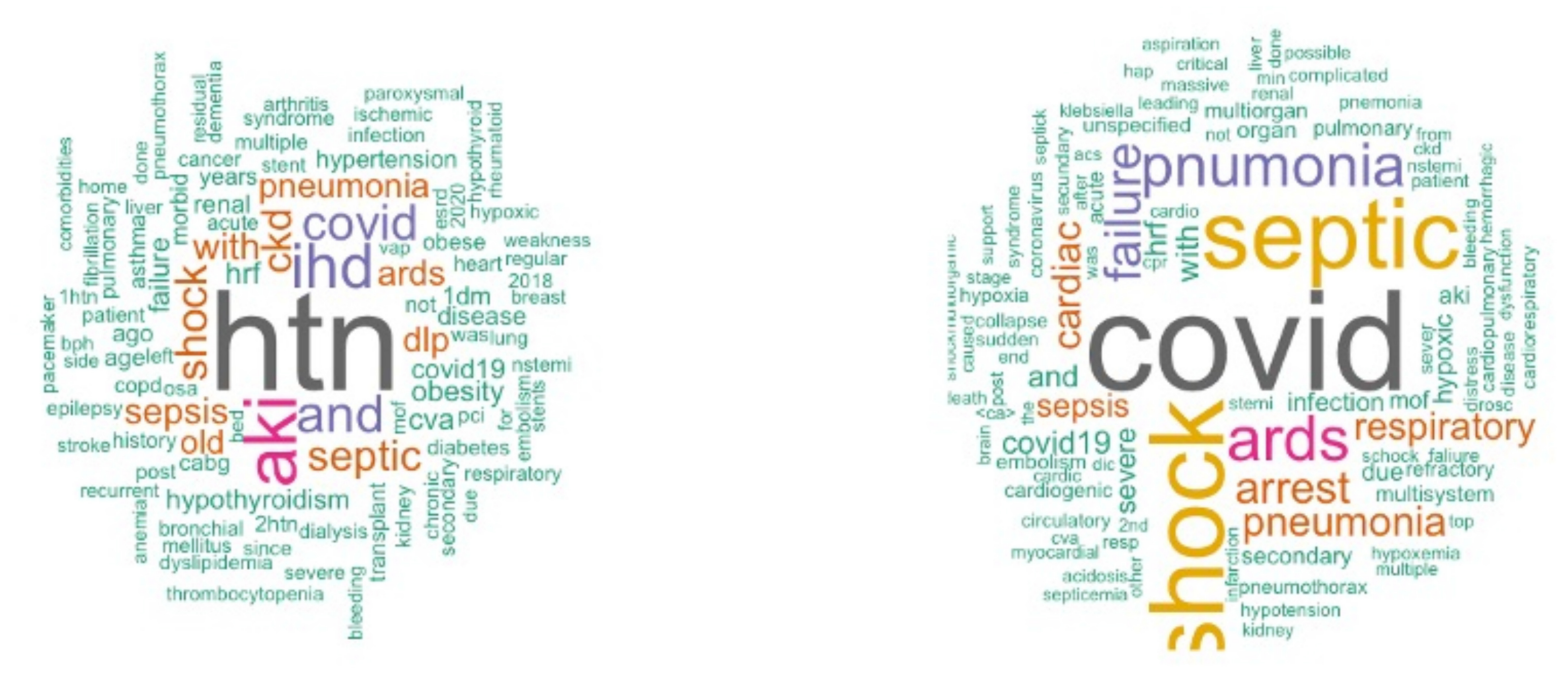
Word cloud plot of death causes and contributing factors.

In order to create BoW, the COD and comorbidity terms were extracted from the EHR in several steps. Starting with a preliminary text analysis using the text mining package (*tm*) and the word cloud generator package (*wordcloud*) in R to extract the most common terms (Figure 1). To create glossary tables, our medical experts validated the extracted terms by reviewing 50–100 EHRs at random. The process was repeated four times to ensure that the majority of the terminologies were covered. This helped identify alternative terminologies and misspelled terms. Tables 1, 2 show the refined list of primary COD and intermediate COD. In accordance with the International Classification of Diseases (11), Table 3 provides twelve general disease categories (GDC), 34 distinct comorbidities, and a risk factor associated with our data. Detailed versions of Tables 1–3, including all potential alternate terms and/or incorrect forms may be requested from the corresponding author.

**Table 1:** Primary causes of death.

**Primary COD (Abbrev.)**	**Alternative terms**
Cardiopulmonary arrest (CPA)	Cardiopulmonary collapse, cardiorespiratory arrest, cardiorespiratory failure, cardiorespiratory collapse, circulatory collapse, asystole
Cardiac arrest (CA)	Cardiogenic shock, cardiovascular collapse, cardiac event, bradycardic arrest, STEMI
Respiratory failure (HRF)	Pulmonary failure, pulmonary arrest, pulmonary dysfunction, hypoxia, hypoxic, hypoxemia, hypoxemic, desaturate
Multiorgan failure (MOF)	MODS, multiple organ dysfunction syndrome, multi organ failure, multiple organ failure, multisystem failure
Hepatic failure (LF)	Liver failure, worsening liver function, hepatic failure
Renal failure (RF)	kidney failure, dialysis, CRRT
Septic shock (SS)	

**Table 2:** Intermediate causes of death.

**Intermediate COD (Abbrev.)**	**Alternative terms**
Acute respiratory distress syndrome (ARDS)	Mechanical ventilation, acute respiratory failure, hypoxic respiratory failure, HRF
Acute kidney failure (AKI)	Acute kidney injury, renal impairment, anuric, hyperkalemia, dialysis
Pulmonary embolism (PE)	DVT collapse, thrombosis
Heart failure (HF)	Rescue PCI, cardiomyopathy, myocarditis
Stroke (ST)	CVA, cerebrovascular accident, failed thrombolysis, hemorrhagic cerebral, subdural, subarachnoid hemorrhage, hge
Pneumothorax (PN)	Tension pneumothorax, hemothorax, hemopneumothorax, hydropneumothorax, pneumoperitoneum, bilateral chest tubes, chest tube
Myocardial infarction (MI)	STEMI, PCI, CCU, ischemic changes, cardiac strain, st elevation, troponin elevated, NStemi
Arrhythmia (AR)	Ventricular fibrillation, VFib, ventricular tachycardia, vtach, rhythm, atrial fibrillation, AF, PAF
Bleeding (BL)	ICH, hematoma, AVM, intracerebral hemorrhage, epistaxis, PRBC, transfusion, melena, upper GI bleeds
Disseminated intravascular coagulation (DI)	DIC
Urinary tract infection (UT)	UTI, urinary tract infection, urosepsis, E.col

**Table 3:** General disease categories (GDC), comorbidities and other risk factors.

**GDC (Abbrev.)**	**Comorbidity/risk factor (Abbrev.)**
Endocrine, nutritional, and metabolic diseases (ENMs)	Diabetes mellitus (DM), thyroid disease (THY), dyslipidemia (DLP), obesity (OB), Addison disease (ADs)
Diseases of the nervous system (DNS)	Stroke (CVA), Parkinson's disease (PD), dementia (DEM), multiple sclerosis (MS), epilepsy (EP), psychiatric disorders (OCD)
Diseases of the circulatory system (DCS)	Hypertension (HTN), anemia (IDA), pulmonary embolism (PE), peripheral vascular disease (PVD), bleeding disorders (BDs)
Cardiovascular system diseases (CVD)	Coronary artery disease (CAD), cardiomyopathy (HCM), valvular heart disease (AVR), heart failure (HF), arrhythmia (AF)
Respiratory diseases (RDs)	Asthma (BA), chronic obstructive pulmonary disease (COPD), lung disease (LD)
GI disorders (GIDs)	Inflammatory bowel disease (IBD), gastroesophageal reflux (GERD), liver disease (LD)
Diseases of the genitourinary (DGS)	Chronic kidney disease (CKD), benign prostatic hyperplasia (BPH)
Autoimmune disorders (ADs)	Rheumatoid arthritis (RA), Immunecompromised (IC) (*a risk factor*)
Ortho disorders (ODs)	Bone disorders (OA)
Infectious diseases (IDs)	HIV-infection (HIV)
Neoplasms (CRC)	Cancer (CA) of any kind
Congenital disorders (CDs)	Down syndrome (DS)

### Developing and applying NLP methods

**Figure 2:**
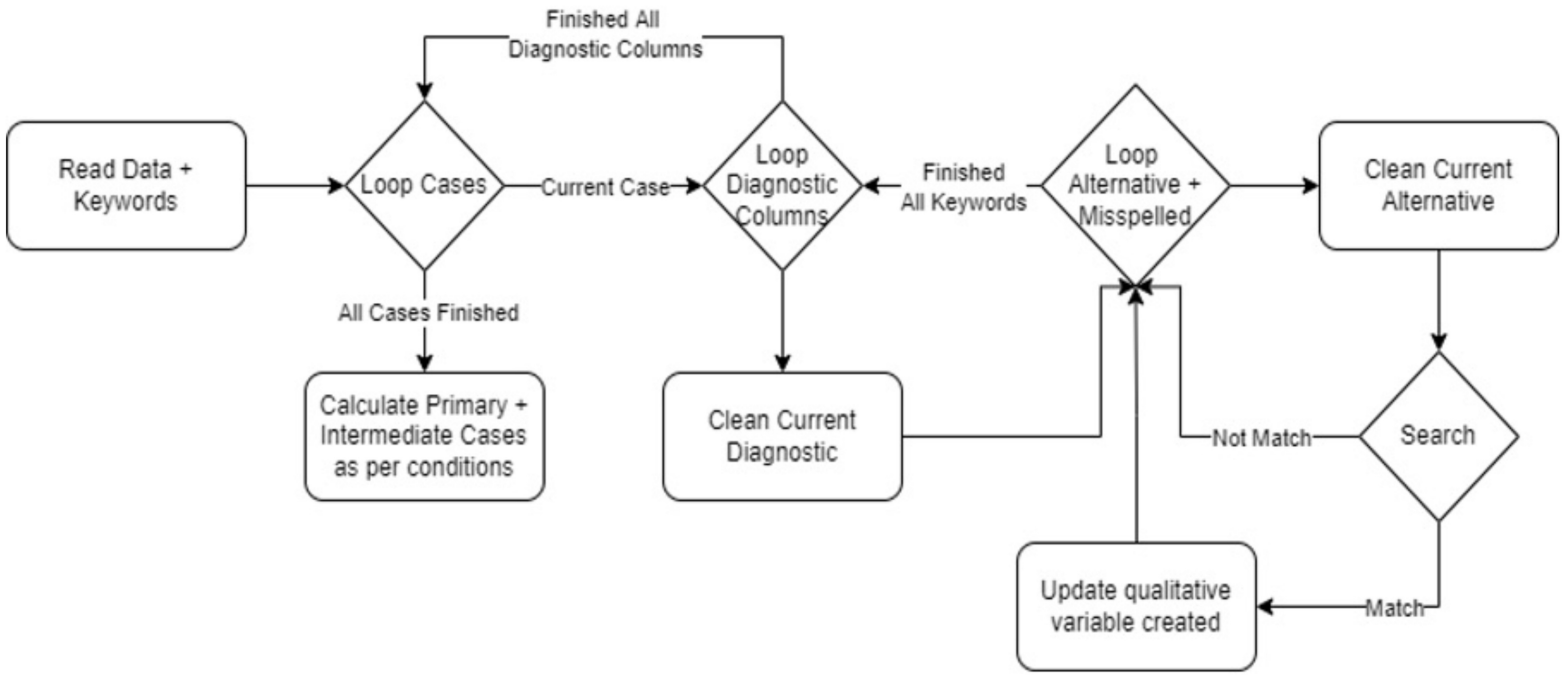
Primary and Intermediate CODs encoding flowchart.

We created an NLP method to identify, extract, and automatically encode natural language from mortality EHRs into structured clinical data. Tables 1, 2 are used as keywords to extract primary and intermediate CODs, while Table 3 presents keywords to extract comorbidities. Method created in Python. Figure 2 shows our algorithm.

**Figure 3:**
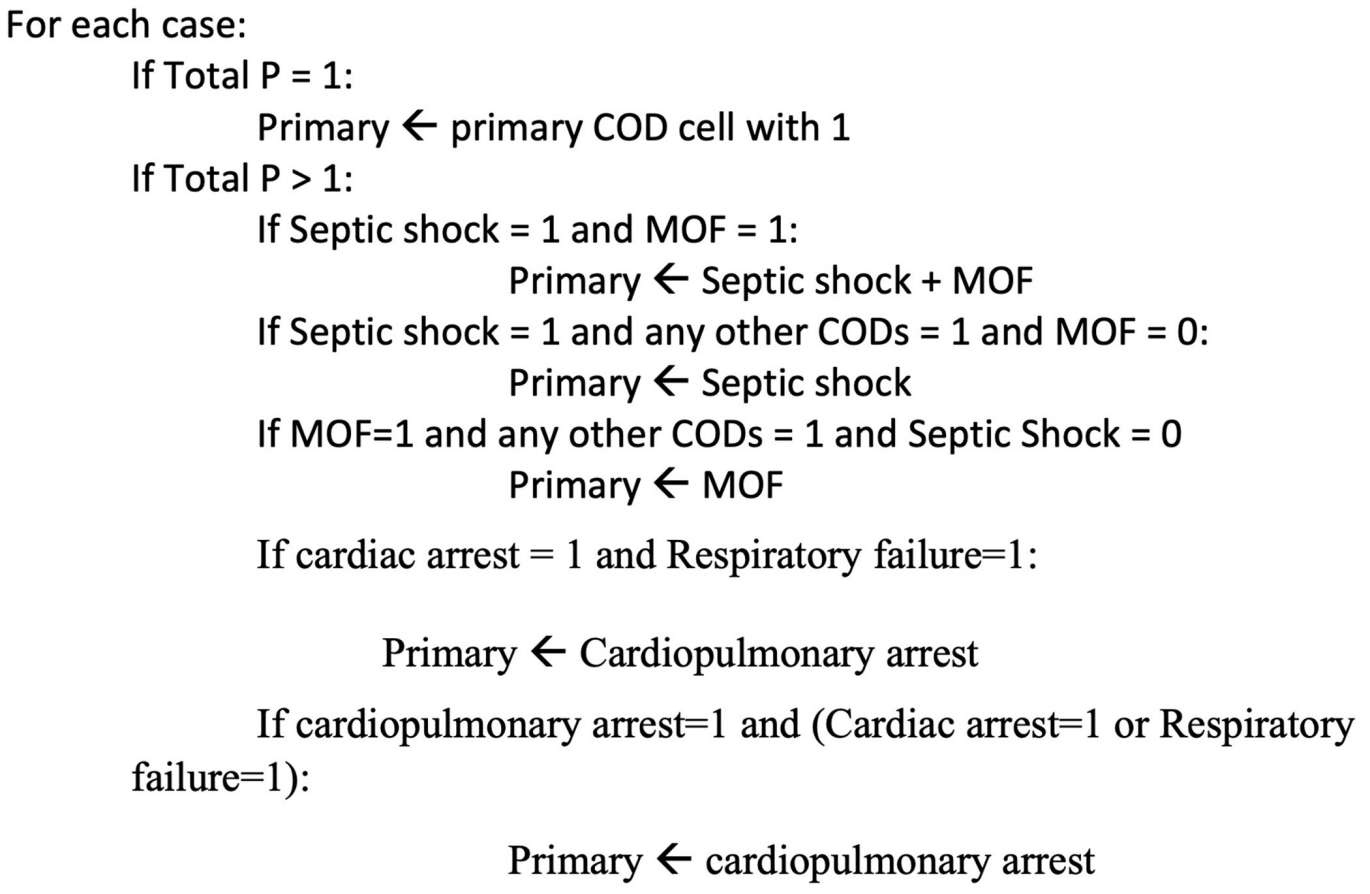
Pseudocode for Primary COD.

In this method, text is stripped of punctuation, special characters, capitalization, stop words, and tokenization. Used EHR variables include cause of death, final diagnosis, brief history, and brief summary. To create a case/COD term occurrence matrix, binary variables must be created for each primary/intermediate COD listed in Tables 1, 2. Initial occurrence matrix setting is zero. CODs or equivalents are compatible with tokens. The case/term occurrence matrix cell is set to 1 upon a match. Every case applies (rows). A COD abbreviation was not mistaken for a term, as PE is not present in hypertensive or hyperthyroid. Negation was also carefully handled; if a term is preceded by a negative or conditional word, it will not match. Exclusion words consist of (no, not, no sign of, non, no history of, no active, no previous medical, not known to have, no indications of, previous condition, old condition). Text format is used to list the final primary and intermediate CODs. The pseudocode used to extract the final primary COD is depicted in Figure 3.

Determining the actual intermediate CODs are handled differently. Multiple intermediate CODs are reported as a group. Our clinicians manually validated and separated the correct outcome to determine which disorders were terminal. A counter matching the extracted causes is also computed to help identify the terminal cause based on the most common causes to cross-check the accuracy of the findings.

The comorbidities for each case are identified using Table 3 in the same manner that CODs are identified. Preprocessed word tokens are extracted from the EHR reason for admission, contributing factors, admission diagnosis and brief summary.

### Data manipulation and analysis

Original EHR mortality data had two sets of variables. First set included seven categorical and quantitative variables. Second set included eight free-text variables. The pdf data sheet was converted to an Excel sheet for data manipulation and cleaning. The second set of data was used to generate 70 variables using Python to determine death causes and comorbidities. During exploratory data analysis, we generated appropriate graphs (bar, pie, boxplots) and summary statistics (mean, median, SD, IQR). Hypothesis tests included Chi-square, TURF, ANOVA, and Kruskal Wallis. Finally, we built our prediction model with a decision tree. SPSS V23 and R were used for the statistical analysis.

## Results

### Overall findings

The majority of the 1,691 anonymous COVID-19 decedents were male 963 (56.9%). The age at death ranges between 19.8 and 103.2 years with 63.8 years (SD 14.4). On the average the duration stay in ICU prior to death was 18.5 days (SD 12.8). Two or more comorbidities were present (mean 2.5, SD 1.9) with hypertension and diabetes mellitus shared among more than half of them (Table 4). Since these patients died in the intensive care unit, COVID-19 pneumonia was mainly the underlying cause of death that resulted in intermediate and thus primary causes of death. COVID-19 pneumonia was detected in 94 percent of cases (1592/1691).

**Table 4:** Demographic and clinical characteristics.

**Variable**	**Summary**	**Count (%)**	**Graph**
Age	Mean (sd): 63.8 (14.4) min ≤ med ≤ max: 19.8 ≤ 64.5 ≤ 103.2 IQR (CV): 20.7 (0.2)	662 distinct values	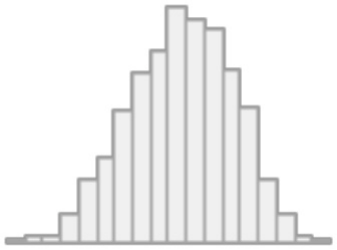
Age group	1. (< 50) years 2. (50–64) years 3. (65+) years	303 (17.9%) 573 (33.9%) 815 (48.2%)	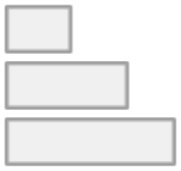
Gender	1. Female 2. Male	728 (43.1%) 963 (56.9%)	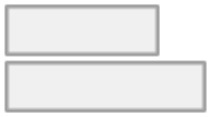
ICU (days)	Mean (sd): 18.5 (12.8) min ≤ med ≤ max: 0 ≤ 16 ≤ 86 IQR (CV): 14 (0.7)	74 distinct values	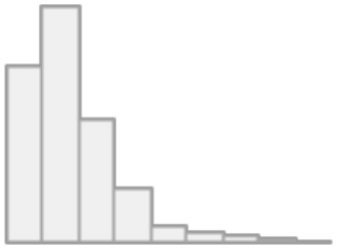
Total comorbidities	Mean (sd): 2.7 (1.9) min ≤ med ≤ max: 0 ≤ 3 ≤ 11 IQR (CV): 3 (0.7)	12 distinct values	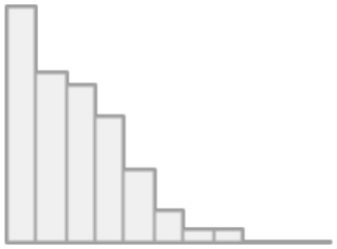
Total comorbidities group	Mean (sd): 2.6 (1.7) min ≤ med ≤ max: 0 ≤ 3 ≤ 6 IQR (CV): 3 (0.6)	0 : 172 (10.8%) 1 : 288 (18.1%) 2 : 333 (20.9%) 3 : 304 (19.1%) 4 : 245 (15.4%) 5 : 143 (9.0%) 6 : 110 (6.9%)	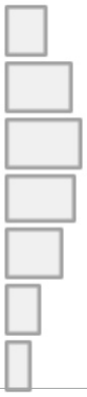

When the mean ICU stay was compared across the three age groups of <50, 50–64, and 65 or more, no significant difference (Table 5) using the ANOVA F-test (*p*-value = 0.903). On the other hand, testing for mean total comorbidities across these three age groups was significant (*p*-value <0.0001), and the Tukey B multiple comparison test reveals significance with three means for groups in homogenous subsets of mean total comorbidities of 1.25, 2.19, and 3.28, respectively.

**Table 5:** Demographic and clinical characteristics by age group.

			**Age group (yrs.)**			
**Variable**	**N**	**Overall**	**Age < 50**	**Age [50-64]**	**Age =65+**	***p*** **-value*^b^***
		***N*** **= 1,691*^a^***	***N*** **= 303*^a^***	***N*** **= 573*^a^***	***N*** **= 815*^a^***	
**Age**	1,691	64 (54, 74)	43 (39, 47)	58 (54, 62)	75 (70, 81)	<0.001
**Gender**	1,691					0.003
Female		728 (43%)	114 (38%)	229 (40%)	385 (47%)	
Male		963 (57%)	189 (62%)	344 (60%)	430 (53%)	
**ICU (days)**	1,691	16 (10, 24)	14 (9, 23)	16 (10, 24)	16 (10, 24)	0.19
**Total comorbidities**	1,596	3 (1, 4)	1 (0, 2)	2 (1, 3)	3 (2, 5)	<0.001
Unknown		95	0	33	62	
**Total group comorbidities**	1,595					<0.001
0		172 (11%)	111 (37%)	61 (11%)	0 (0%)	
1		288 (18%)	85 (28%)	122 (23%)	81 (11%)	
2		333 (21%)	59 (19%)	136 (25%)	138 (18%)	
3		304 (19%)	30 (9.9%)	99 (18%)	175 (23%)	
4		245 (15%)	12 (4.0%)	72 (13%)	161 (21%)	
5		143 (9.0%)	2 (0.7%)	32 (5.9%)	109 (14%)	
6		110 (6.9%)	4 (1.3%)	18 (3.3%)	88 (12%)	
Unknown		96	0	33	63	

a Median (IQR) or Frequency (%).

b Kruskal-Wallis rank sum test; Pearson's Chi-squared test.

### Clinical characteristics and common causes of death among COVID-19 patients

**Figure 4:**
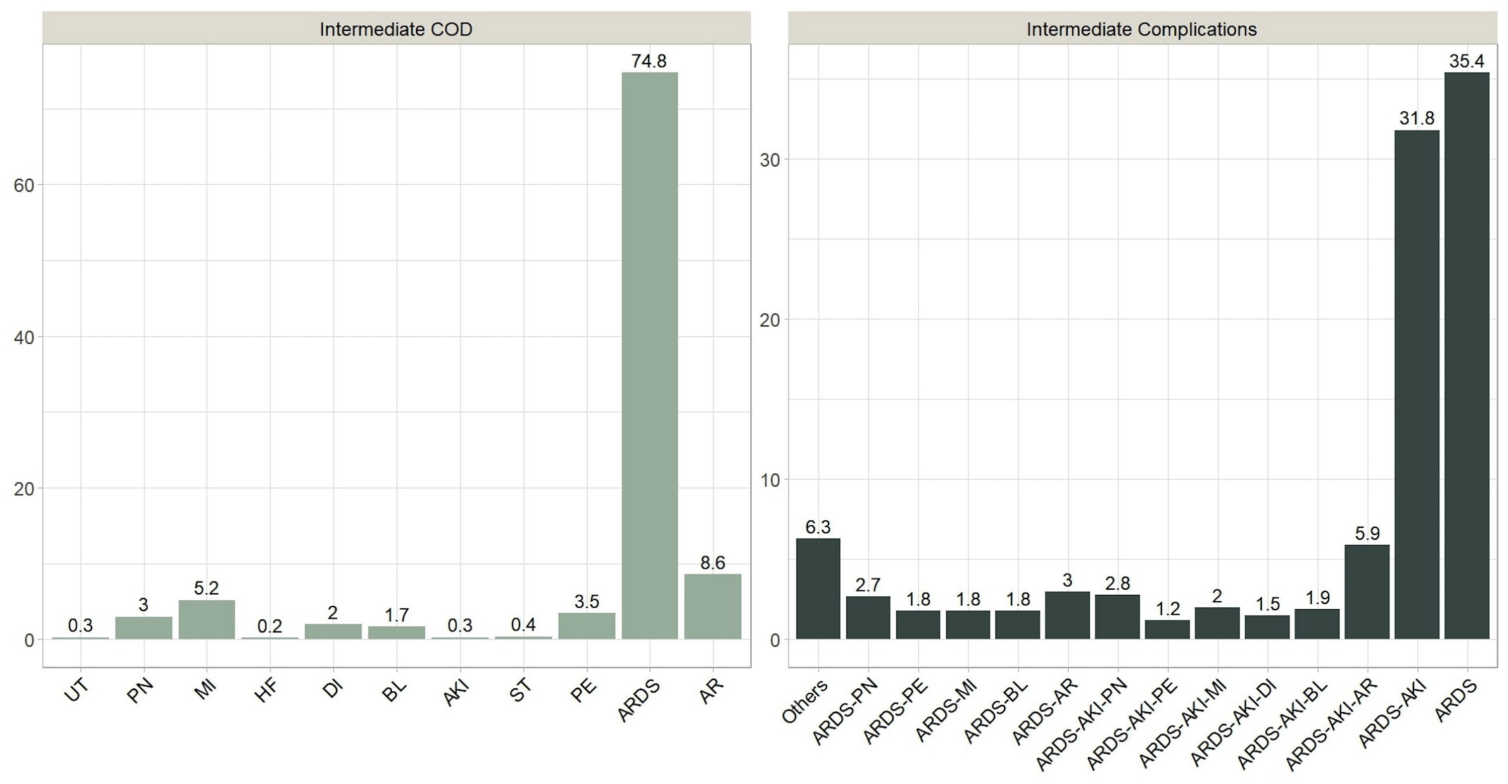
Combined intermediate complications and terminal Intermediate COD.

We identified primary and secondary causes of death. Septic shock was the primary COD in 667 patients (39.4%), followed by cardiopulmonary arrest 304 (18.0%), respiratory failure 235 (13.9%), and cardiac arrest 180 (10.6%). The percentages of cases with (septic shock & MOF), MOF, and renal failure were 135 (8.0%), 125 (7.4), and 44 (2.6%), respectively. Hepatic failure occurred in only one case and thus ignored from further analysis. On the other hand, ARDS was one of the main reasons for ICU admissions and was reported in all deaths. Numerous cases were reported in which a combination of intermediate death complications occurred. These cases were thoroughly examined by our physicians to determine which terminal complication is more likely to be classified as the intermediate COD. It was found that around 75% of these decedents had ARDS as an intermediate COD, while the remaining 25% had intermediate COD other than ARDS. Among the other causes are AKI, AR, BL, DI, HF, MI, PE, PN, ST, and UT. The frequency distribution of intermediate combined complications along with the frequency distribution of the terminal complication leading to intermediate COD are shown in Figure 4. Table 6 shows the count and percentage of counts for primary and intermediate causes, as well as the column percentages for primary causes. While ARDS is the most prevalent intermediate COD regardless of primary cause, AR and MI disorders were significantly (7.2–14.4%) linked with cardiac arrest and MOF.

**Table 6:** Primary by intermediate causes of death.

	**Primary COD count (%)**							
**Inter-mediate COD**	**SS**	**CPA**	**HRF**	**CA**	**SS + MOF**	**MOF**	**RF**	**Total**
**AR**	53 (7.9)	26 (8.6)	11 (4.7)	26 (14.4)	14 (10.4)	12 (9.6)	3 (6.8)	145 (8.6)
**ARDS**	523 (78.4)	220 (72.4)	194 (82.6)	117 (65)	94 (69.6)	8 (64.8)	35 (79.5)	1,265 (74.8)
**BL**	12 (1.8)	3 (1)	5 (2.1)	3 (1.7)	1 (0.7)	5 (4)	0 (0)	29 (1.7)
**DI**	14 (2.1)	2 (0.7)	0 (0)	1 (0.6)	11 (8.1)	4 (3.2)	1 (2.3)	33 (2)
**HF**	0 (0)	2 (0.7)	0 (0)	1 (0.6)	0 (0)	1 (0.8)	0 (0)	4 (0.2)
**MI**	23 (3.4)	26 (8.6)	4 (1.7)	21 (11.7)	5 (3.7)	9 (7.2)	0 (0)	88 (5.2)
**PE**	14 (2.1)	16 (5.3)	12 (5.1)	7 (3.9)	5 (3.7)	5 (4)	1 (2.3)	60 (3.5)
**PN**	22 (3.3)	8 (2.6)	7 (3)	3 (1.7)	2 (1.5)	6 (4.8)	3 (6.8)	51 (3)
**ST**	2 (0.3)	1 (0.3)	1 (0.4)	1 (0.6)	1 (0.7)	0 (0)	0 (0)	6 (0.4)
**UT**	2 (0.3)	0 (0)	1 (0.4)	0(0)	1 (0.7)	1(0.8)	0 (0)	5 (0.3)
Total	667 (100)	304 (100)	235 (100)	180 (100)	135 (100)	125 (100)	44 (100)	1,691 (100)

**Figure 5:**
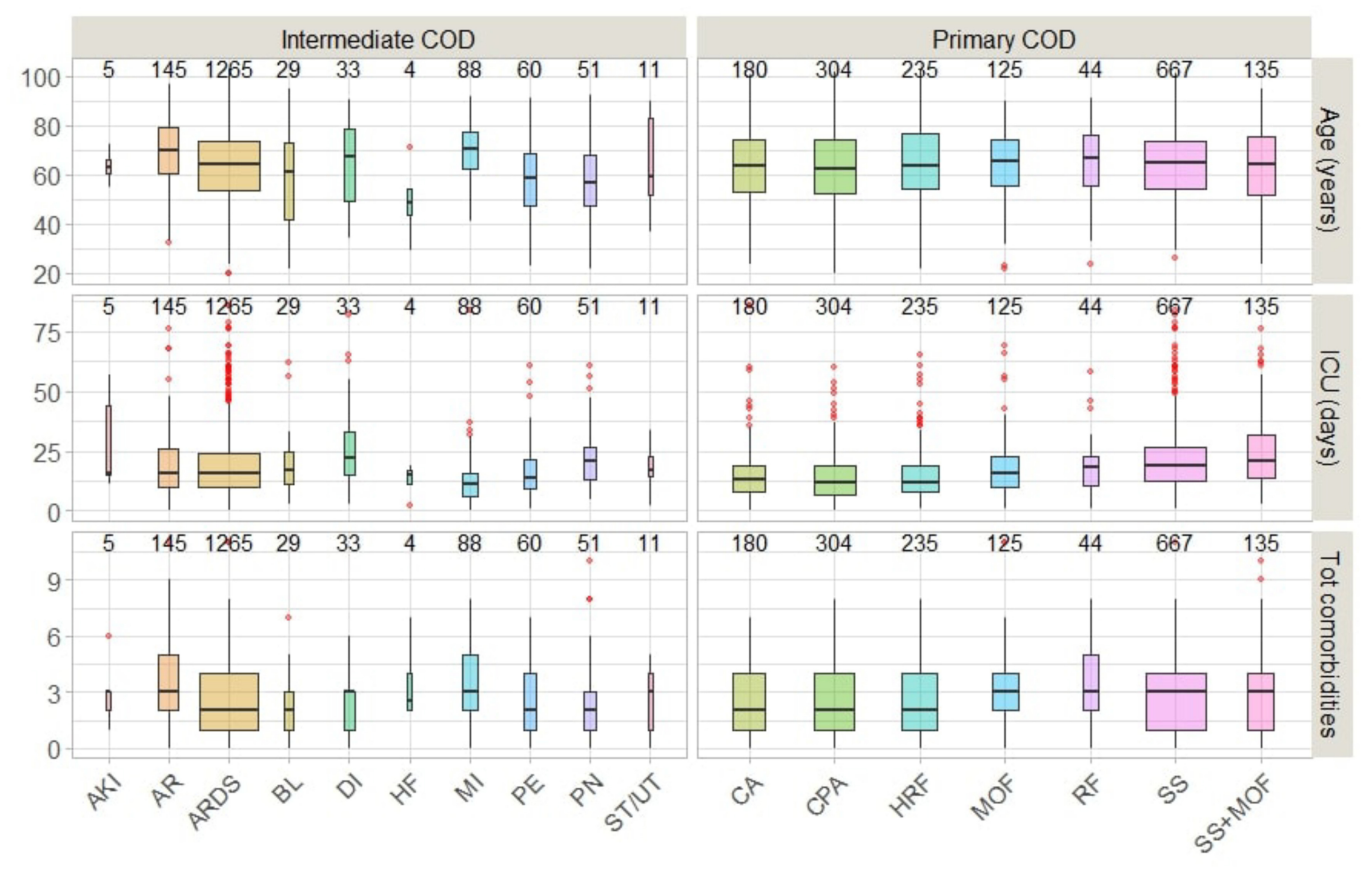
Primary and Intermediate CODs distribution by age and clinical characteristics.

Age distribution appears to be similar by primary COD, with a median age at death of 64.5 years and an interquartile range (IQR = 20.7). However, a few young patients, approximately the age of 20, died because of MOF or renal failure (Figure 5). The median length of stay in the ICU prior to death was approximately 16 days overall but was significantly longer (~ 20 days) for those who died of septic shock or (septic shock + MOF). Patients who died because of MOF had an average of three or more comorbidities. Those who died of renal failure and (septic shock + MOF) died in a manner like that described above.

Those who died because of AR, DI, or MI had the highest average age (70 years) and total comorbidities (3 or more), as well as the shortest average stay in the ICU. Patients who died of HF were younger (average age 50 years) and had more than two comorbidities, with an ICU stay of < 20 days. We also noticed the sequences of (MI → septic shock) and (PE → respiratory failure) were associate with 4 or above comorbidities on the average.

### Exploring the relationship between comorbidities and causes of death

The following is a list of the comorbidities of dead patients in this study. Hypertension (57%) is the most common condition, followed by diabetes (52%), coronary artery disease (23%), and chronic renal disease (14%). 12% for each arrhythmia and dyslipidemia, cancer (11%), Rheumatoid arthritis (10%), obesity (9%), thyroid disease (8%), stroke (7%), pulmonary embolism (5%), asthma (4%), valvular heart disease (4%), bleeding disorders (4%), and 3% for each COPD and dementia. The remaining comorbidities with < 3% reported incidence include Anemia, heart failure, prostate hyperplasia, liver disease, epilepsy, cardiomyopathy, peripheral vascular disease, lung disease, psychiatric disorders, osteoporosis, multiple sclerosis, down syndrome, Parkinson's disease, inflammatory bowel disease, gastroesophageal reflux disease, Addison disease, and HIV infection.

Next, we present the results of the Total Unduplicated Reach and Frequency (TURF) method. TURF is a popular statistical technique in market research that ranks product combinations according to the number of customers who favor them (12). In this study, we applied the method in a clinical setting, treating comorbidities and patients as products and people. The goal is to determine the most likely disease combinations that these patients share. The analysis traverses all possible combinations of comorbidities and records two statistics for each: reach and frequency. The reach is the percentage of individuals who exhibit at least one comorbidity in a given combination, and the frequency is the total number of times comorbidities are exhibited in a given combination. We tested the method for all comorbidities listed in Table 3 and a range of reach values. Table 7 provides a summary of the ideal choices according to the number of diseases (Size). For instance, the optimal combination of four comorbidities has a 73 percent success rate with RA, cancer, DM, and HTN. This indicates that seventy-three percent of the patients had at least one of the conditions (rheumatological disorders, cancer, DM, HTN). If Diabetes and High Blood Pressure were eliminated from the analysis due to their high prevalence and we wanted to evaluate other possible combinations of diseases, the one with the highest prevalence was (obesity, CAD, Cancer, RA) with 43.6%.

**Table 7:** Best reach and frequency by group size.

	**Size**	**Reach**	**Cases %**	**Count**	**Responses %**
ADDED: HTN	1	962	56.9	962	27.7
ADDED: DM	2	1,139	67.4	1,834	52.9
KEPT: HTN					
ADDED: Cancer	3	1,195	70.7	2,026	58.4
KEPT: DM, HTN					
ADDED: RA	4	1,234	73.0	2,196	63.3
KEPT: Cancer, DM, HTN					
ADDED: AF	5	1,266	74.9	2,402	69.3
KEPT: Cancer, DM, HTN, RA					
ADDED: Obesity	6	1,296	76.6	2,550	73.5
KEPT: AF, Cancer, DM, HTN, RA					

**Figure 6:**
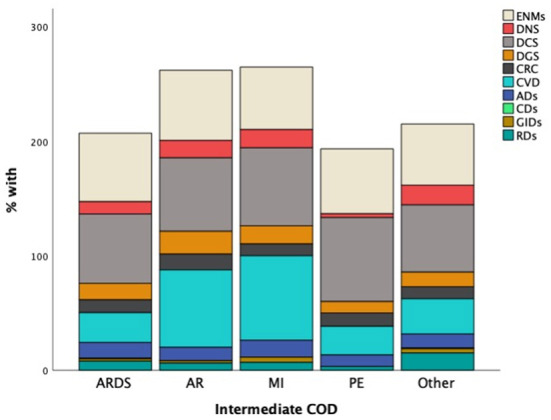
Proportions of general disease categories by Intermediate COD.

When we looked at the general disease classification frequencies, we found that over 60% of the patients had circulatory (DCS) and endocrine (ENMS) disorders, one-third had cardiovascular diseases (CVD), and the remaining categories (RDs, CRC, DNS, ADs, DGS) varied from 8 to 15%. In compared to patients who died of ARDS/PE/Other, approximately 65 percent of patients who died of MI or AR had cardiovascular illnesses (Figure 6). Those who die from ARDS, on the other hand, usually have endocrine or circulatory system problems. Nervous system diseases were the least common among the PE dead. With chi-square test findings of (175.5, *p*-value 0.001) and (12.2, *p*-value = 0.016), the circulatory and nervous systems had the most significant association with intermediate COD.

### Predicting death due to ARDS or other causes

**Figure 7:**
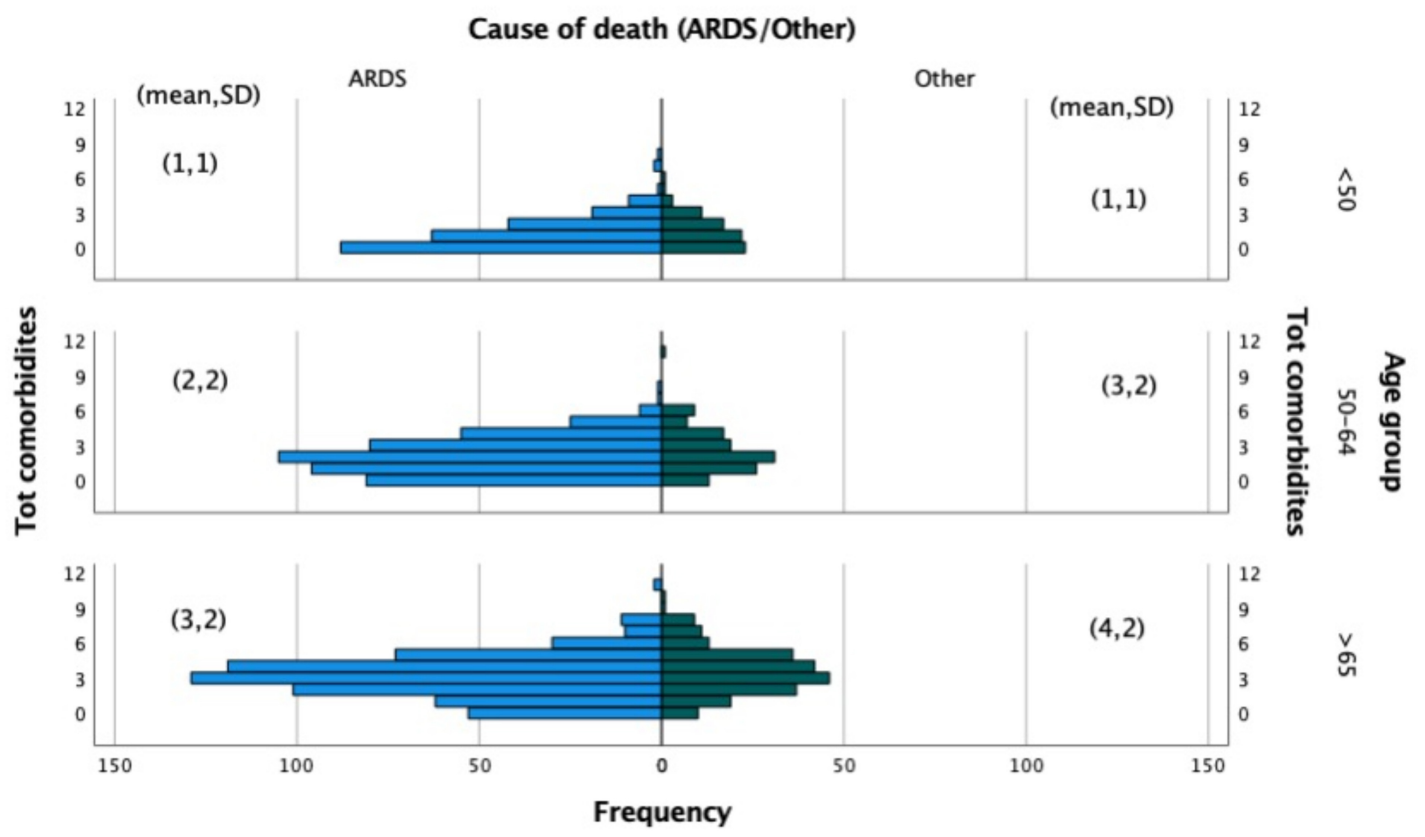
Total comorbidities by age group due to ARDS or other causes of death.

The total comorbidities distribution by age group of COVID-19 deaths due to ARDS or other cause is displayed in Figure 7. Patients under the age of 50 have a similar comorbidity distribution, with an average of one disease. Two comorbidities were found on average per age group (50–64) with more variation among those who died from causes other than ARDS. In contrast, older patients (age >65) who died from causes other than ARDS have an average of four comorbidities, compared to three for the other group who died mainly from ARDS.

**Figure 8:**
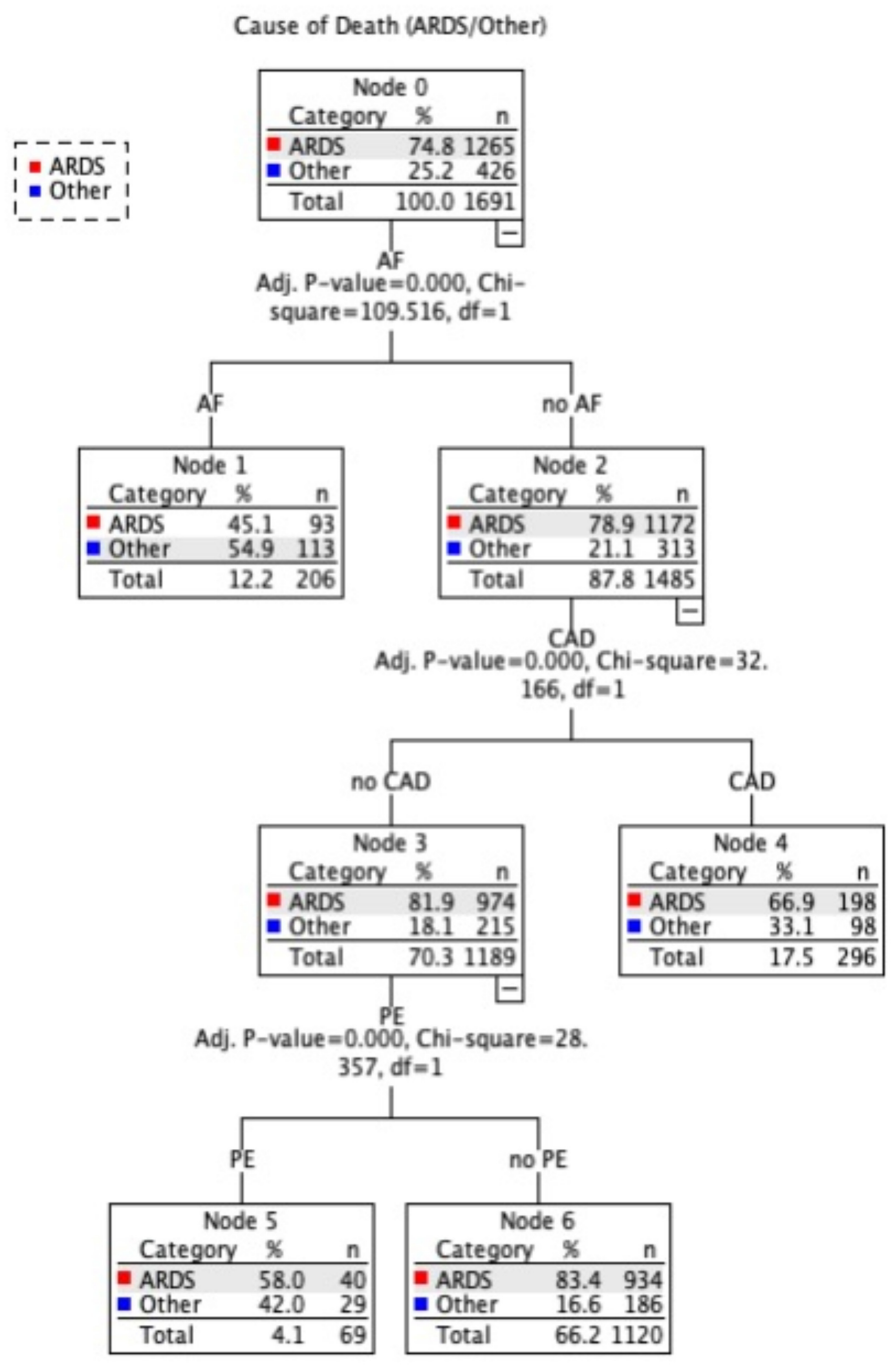
Decision Tree prediction model of ARDS/Other causes of death.

In this section, we used decision tree (DT) to determine the most parsimonious predictors of intermediate COD among COVID-19 patients in intensive care units. Decision trees learn to divide data into smaller and smaller categories to forecast the goal. The test is represented by a node, while the numerous outcomes are represented by edges. The dividing process is repeated until no further gains can be obtained or a preset rule is reached. Three common decision tree techniques include classification and regression tree (CART), chi-squared automatic interaction detection (CHAID), and quick unbiased efficient statistical tree (QUIEST). For mathematical explanations and performance comparisons of these DT approaches, see Lin et al. (13). Figure 8 illustrates the results of the QUEST model, which demonstrate that the existence of an arrhythmia (AF) was the best indicator of the intermediate cause (ARDS/Other). Patients with AF are more likely to have a cause other than ARDS (54.9%). Node 1 is considered a terminal node for predicting a cause of death other than ARDS since no child nodes was found below it. In patients without AF, on the other hand, CAD was the second-best predictor of (ARDS/Other). In patients without AF but with CAD, the terminal Node 3 predicted 66.9 ARDS vs. 33.1% for other causes. PE is an additional predictor in the model for patients who do not have AF or CAD. ARDS is the main intermediate COD in this group, accounting for over 83% of patients without PE and 58% of patients with PE who died from ARDS. The risk and classification tables allow for a quick evaluation of the model's performance. The risk of misclassifying the cause of death is estimated to be 0.272 (or 27.2%), which is consistent with the results of the classification table, which show that 76% of causes of death are correctly classified.

## Discussion

We used Machine learning NLP to extract clinical data and causes of death from EHRs for COVID-19 patients at Jaber Hospital in Kuwait. Consistency and completeness issues with the text data in these records made extraction difficult. During the pandemic, Jaber hospital was restricted to COVID-19 admissions, with most critical cases transferred from other hospitals. Many patient records were incomplete due to patients being transferred from district hospitals where their original medical records were kept. Machine learning and big data analytics have been used to investigate disease-related prognostic factors (14).

Several clinical characteristics have been linked to COVID-19 mortality. Age, gender, comorbidities, ICU stay, and disease severity are all factors. Increased proportions of 65-year-olds or older led to a significant age-mortality association (15, 16). Males were more likely to die from COVID-19 (17, 18). More than double the number of death patients had two or more comorbidities, according to Ayed et al. (17). Combining old age and comorbidities was also a factor in death (19) and survival time (20). On the other hand, Zhou et al. (21) reported a median (IQR) time of 18.5 (15–22) days from onset of symptoms to death. In our study, 815 (48%) of 1691 deceased ICU COVID-19 patients were over 65, men were more prevalent (56.9 vs. 43.1%), patients with two or more comorbidities accounted for 52% of cases, and the mean (SD) survival time to death was 18.5 (12.8) days. Hypertension and diabetes accounted for more than half of all cases in this study. This confirms prior research (17, 22–24). In COVID-19 patients, cardiovascular disease and secondary infections increase disease severity and mortality (15, 25, 26). Circulatory and cardiovascular diseases account for 61.6 and 32.5% of these patients, respectively; HIV-infections are rare. COVID-19 patients had a higher incidence of kidney and heart disease, and myocardium damage reduced survival (16, 27, 28).

Previous research on comorbidities and death causes has linked dysfunction to mortality (17, 29). In this study, decedents with MOF and renal failure averaged three or more comorbidities. Septic shock was the leading primary cause, accounting for 667 deaths (39.4%), followed by cardiopulmonary arrest (304 deaths, 18%), respiratory failure (235 deaths, 13.9%), and cardiac arrest (180 deaths, 10.6%). The most common intermediate COD, on the other hand, was ARDS (1265, 74.8%). We also found 849 (50.2%) cases of sepsis. Other findings (21) revealed that sepsis was the leading cause of death (59%) among the 54 pandemic deaths, followed by respiratory failure (54%), ARDS (31%), heart failure (23%), and septic shock (20%).

Acute respiratory distress syndrome (ARDS) is a severe COVID-19 consequence. Patients with moderate-to-severe ARDS require invasive mechanical ventilation and intensive medical therapy (30, 31). ARDS was one of the most common reasons for ICU hospitalizations, as it was recorded in 81.8% of ICU survivors and all fatalities (32). This is also demonstrated in our data, as all patients were admitted to the intensive care unit, and ARDS was a common morbid consequence. However, complications other than ARDS were deemed the predominant intermediate COD in 25% of the cases (Figure 4). As a result, we employed decision trees to forecast the most significant contributing factors to intermediate COD, namely ARDS or Other cause. “Other” denotes a complication associated with AKI, AR, BL, DI, HF, MI, PE, PN, ST, or UT. We encountered only three significant predictors, namely arrhythmia (AF), coronary artery disease (CAD), and pulmonary embolism (PE). Patients with AF were more likely to have an etiology other than ARDS. According to Elezkurtaj et al. (33), the majority of decedents died from COVID-19, with preexisting health conditions and comorbidities only contributing to the mechanism of death. We agree because, among the many variables examined in this study, only a few contributing factors were found to be significant with intermediate COD.

### Strengths, limitations, and future work

The dynamic nature of the method, its usability, and its potential to maintain self-control all contribute to its strength. In addition, the sampled data span both significant pandemic waves and death peaks, accounting for 70% of the total reported COVID-19 fatality cases in Kuwait. The death rate drastically decreased after then. Therefore, our sample represents the population under consideration to a high degree of accuracy. Nevertheless, our study has several limitations. First, there is a chance of selection or referral bias as the research was conducted at a single location, i.e., Jaber Hospital. Second, the lack of information extracted from the inadequate documentation of the patient records. The absence of a symptom (such as obesity, smoking, etc.) does not necessarily suggest that a patient is symptom-free. Thirdly, patients were typically transferred late in the course of their disease, and their medical records lacked vital medical history information. Such discrepancies in clinical data may result in information bias that contributes to a decrease in model precision.

Future studies could potentially investigate the impact of vaccines on the time to death, provide survival time estimates by cause of death, and perform spatiotemporal analyses of transferable patients. Knowing the COVID-19 death rate and patient survival rate can help risk management experts. COVID-19 or its evolving variants can be avoided, and strategies can be used to slow their spread.

## Conclusion

We employ self-developed natural language processing (NLP) to automate the extraction of causes of death and comorbidities from the EHRs of COVID-19 decedents from the beginning of the pandemic through all major pandemic waves in this study. We structured the acquired text data and used it to conduct additional research.

We analyzed the demographic, clinical, and causes of death data for 1,691 ICU patients and discovered that the most common primary causes of death, which were documented in 54.8% of cases, were infection-related and included septic shock or sepsis-related multi-organ failure. The second most common cause of death was respiratory failure or cardiopulmonary arrest, which were documented in 32.2% of cases. Furthermore, cardiac arrest and renal failure account for 10.6 and 2.6% of all deaths, respectively. ARDS, on the other hand, was the most common cause of mortality in the intermediate stage. Arrhythmia (AF) was revealed to be the strongest predictor of intermediate cause (ARDS/Other) using machine learning decision tree analysis.

We recommend structuring the EHR with well-defined sections and providing menu-driven options for reporting causes of death and comorbidities to minimize misspellings or incorrect forms. Comprehensive assessment and user guidance are required for standards to be effectively integrated into EHR systems.

## Data availability statement

The raw data supporting the conclusions of this article will be made available by the authors, without undue reservation.

## Ethics statement

The studies involving human participants were reviewed and approved by Ethical Review Committee (ERC) at Kuwait Ministry of Health (No. 1529/2020). Written informed consent for participation was not required for this study in accordance with the national legislation and the institutional requirements.

## Author contributions

SB conceived and gained ethical approval for the project. SB, AB, and YA participated in the retrieval, processing, and purification of data. AB and YA developed the clinical concepts, played a key role in establishing the data extraction clinical criteria, and validations. SB and AA created both the method and the programming. SB and SA carried out statistical analysis and produce visuals. SB, AA, and SA contributed to the paper's drafting. All authors have reviewed, offered comments, and approved the submission of the work.

## Conflict of interest

The authors declare that the research was conducted in the absence of any commercial or financial relationships that could be construed as a potential conflict of interest.

## Publisher's note

All claims expressed in this article are solely those of the authors and do not necessarily represent those of their affiliated organizations, or those of the publisher, the editors and the reviewers. Any product that may be evaluated in this article, or claim that may be made by its manufacturer, is not guaranteed or endorsed by the publisher.
